# The Mechanism of Asparagine Endopeptidase in the Progression of Malignant Tumors: A Review

**DOI:** 10.3390/cells10051153

**Published:** 2021-05-10

**Authors:** Wenrui Zhang, Yingying Lin

**Affiliations:** Department of Neurosurgery, Ren Ji Hospital, School of Medicine, Shanghai JiaoTong University, Shanghai 200127, China; wwwrzhang@163.com

**Keywords:** asparagine endopeptidase, glioblastoma, breast cancer, gastric cancer, epithelial ovarian cancer

## Abstract

Asparagine endopeptidase (AEP), also called legumain, is currently the only known cysteine protease that specifically cleaves peptide bonds in asparaginyl residue in the mammalian genome. Since 2003, AEP has been reported to be widely expressed in a variety of carcinomas and is considered a potential therapeutic target. In the following years, researchers intensively investigated the substrates of AEP and the mechanism of AEP in partial tumors. With the identification of substrate proteins such as P53, integrin αvβ3, MMP-2, and MMP-9, the biochemical mechanism of AEP in carcinomas is also more precise. This review will clarify the probable mechanisms of AEP in the progression of breast carcinoma, glioblastoma, gastric carcinoma, and epithelial ovarian carcinoma. This review will also discuss the feasibility of targeted therapy with AEP inhibitor (AEPI) in these carcinomas.

## 1. Introduction

### 1.1. Structure and Classification of AEP

**Figure 1:**
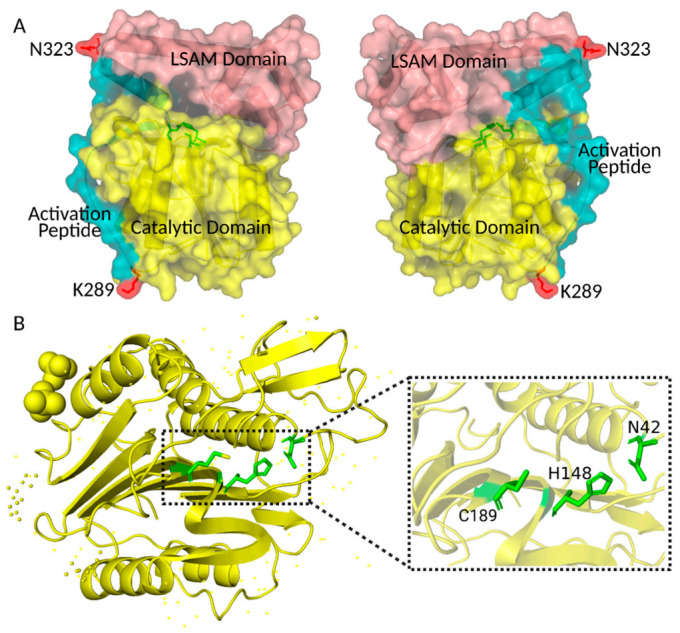
Crystal structure of human pro-AEP and AEP. (**A**) Crystal structure of human pro-AEP. The caspase-like catalytic domain is shown in yellow, the Activation Peptide in blue and the LSAM domain in pink; the C-terminal processing sites K289 and N323 are shown in red. (**B**) Crystal structure of human AEP. The active site residues (Asn42, His148, and Cys189) of the catalytic domain are shown in green. The figure was created using PYMOL.

Asparagine endopeptidase (AEP) is a cysteine protease that was initially discovered in leguminous seeds in the early 1980s and was first isolated and identified in mammals in 1997 [1,2]. AEP is named after its strict specificity for cleavage in asparagine residues, and it is also known as legumain (LGMN) due to its existence in many leguminous seeds. The gene encoding human AEP is located on chromosome 14 at the locus 14q32.12, encoding for a pro-enzyme of 433 amino acids. The sequence of human AEP shares a homology of 83% with murine protein [3,4]. The crystal structure (three-dimensional structure) of human pro-AEP consists of a caspase-like catalytic domain, an activation peptide (AP), and a legumain stabilization and activity modulation (LSAM) domain [5,6] (Figure 1). The LSAM domain can stabilize pro-AEP at neutral pH by triggering an electrostatically encoded stability switch (ESS) that is localized near the catalytic domain. Active site residues His^148^, Cys^189^, and Asn^42^ of the catalytic domain are in close spatial proximity to each other, and are essential for the enzymatic activity of AEP [5,6] (Figure 1). In addition, because of the highly conserved His^148^- Gly-spacer-Ala-Cys^189^ motif, AEP was classified as a member of the C13 family of cysteine proteases (EC 3.4.22.34), suggesting an evolutionary relationship with the caspases, the bacterial proteases gingipain and clostripain, and separase [7].

### 1.2. Localization, Activation, and Inhibition of AEP

AEP predominantly exists in the late endosomes and lysosomes. The process of activation in vitro is well understood. Pro-AEP is stable at neutral pH, while an acidic pH shift can trigger the autocatalytic processing of pro-AEP. Pro-AEP (53 kDa) is autoactivated at pH 4.5~5.5 into an intermediate form (46/47 kDa) through self-cleavage at Asn^323^ (pH ≤ 5.5) and Asp^25^ or Asp^21^ (pH ≤ 4.5), respectively. As cleavage at Asn^323^ site of the C-terminal proved critical for active AEP, cleavage at the N-terminal is not essential for activation [8]. The intermediate form of AEP (46/47 kDa) has further cleavages from other cysteine proteases, generating mature AEP of 36 kDa after cleavage of the C-terminal Asp^303/309^ (pH ≤ 4.0) [9,10]. Interestingly, both the intermediate form (46/47 kDa) and the mature form (36 kDa) of AEP possess similar enzymatic activities, and both of them can be inhibited by the endogenous proteinase [10]. There are several cysteine protease inhibitors in mammals; however, only the family 2 cystatins were able to inhibit AEP. The family 2 cystatins include cystatin C, E/M, and F. Cystatin E/M is the most powerful inhibitor for regulating active AEP through substrate competition in mammals [11,12,13,14]. At near-neutral pH, AEP possesses ligase activity. Autocatalytic processing at the Asn^323^ site is reversible in mammals, and therefore, an already proteolytically activated AEP can be converted back into the latent zymogen state through auto-ligation [15]. The lysosomal pH is 5–4.5 in late endosomes, but there is no direct evidence that AEP is activated in late lysosomes in vivo [16].

Among the synthetic substances, the most effective AEP inhibitor is aza-Asn epoxide, a small molecule compound with a specific effect [17]. AEPI is the most promising chemotherapeutic agent for targeting AEP.

### 1.3. Physiological or Pathological Roles of AEP in Mammals

AEP-deficient (LGMN-/-) mice are normally born with no distinct anatomical or morphological abnormalities, but experience significant loss of body weight. Disruption of the AEP gene led to the outcome of enlarged lysosomes, deficient processing of lysosomal cathepsins and toll-like receptors, kidney failure, and hemophagocytic syndrome, thus demonstrating its significant role in in mammalian physiology [18,19,20].

In the mammalian immune system, AEP is localized in antigen-presenting cells (APCs), such as B cells and dendritic cells (DCs). In B cells, AEP involves processing self and foreign proteins for presentation of the class II major histocompatibility complex (MHC II) on the surface of T cells, thus helping in the development of immune tolerance [21,22]. The loss of this function is related to multiple sclerosis; exaggerated AEP protease activity destroys the myelin basic protein (MBP) peptides, leading to the failure of immune tolerance in the thymus against MBP [23]. In DCs, AEP also plays an indispensable role in activating toll-like receptor 9, which is critical for full cytokine production [19,24]. Recently, AEP was reported to regulate the stability of FOXP3, a transcription factor that controls the immunosuppressive program in CD4(+) T cells. Blocking AEP imparted a regulatory function in inducible regulatory T cells [25].

The roles of AEP have also been reported in other systems of mammals. In normal human bone marrow, AEP was called Osteoclast Inhibitory peptide 2 (OIP-2), as it plays roles in osteoclast formation and bone resorption by regulating the differentiation fate of human bone marrow stromal cells (hBMSCs). Dysfunction of AEP is associated with bone mass reduction in postmenopausal osteoporosis [26,27]. In the mammalian urinary system, AEP is abundant in the kidneys, especially in the renal proximal tubular cells. Degradation of fibronectin by AEP among renal proximal tubular cells led to extracellular matrix (ECM) remodeling. The dysregulation of AEP is thought to be the pathogenesis of renal interstitial fibrosis [2,18]. The involvement of AEP in the remodeling of the ECM is not restricted to the kidneys. In other inflammations, such as idiopathic pulmonary fibrosis, liver fibrosis, atherosclerosis, and pancreatitis, AEP was considered to be an effective therapeutic target based on this role [28,29,30,31,32]. However, this hypothesis needs more evidence in order to be proven. In the central nervous system, AEP is associated with neurodegenerative diseases (NDGs), including Alzheimer’s disease, stroke, ischemia, amyotrophic lateral sclerosis (ALS), and multiple sclerosis. AEP is involved in autophagy in lysosomes and the cleavage of proteins involved in NDGs, such as α-synuclein, TDP-43, tau, and amyloid precursor protein (APP) [33,34,35,36,37,38,39].

As a highly specific protease, AEP also has a place in cancer. In 2003, Liu C. et al. proved the overexpression of AEP in human solid tumors, including breast carcinoma, colon carcinoma, lung carcinoma, prostate carcinoma, ovarian carcinoma, lymphoma, and melanoma, through immunohistochemistry. In vitro and in vivo experiments demonstrated AEP as a potential target for tumor therapy [40]. This report has triggered a switch for life science researchers toward exploring the role of AEP in multiple tumor types. In the following decade, highly expressed AEP in gastric carcinoma [41,42] and hepatocellular carcinoma [42] were successively reported.

A published review summarized the expression of AEP in multiple tumors; also, its summary of targeted therapies for AEP highlighted the potential of AEP as a therapeutic target for tumors [43]. Overall, the tumor-promoting role of AEP in cancer is well established. As the biochemical mechanisms of AEP have been studied in depth, there are subtle differences in the roles played by AEP in different tumors.

In this review, we focus on four carcinomas that have been studied to a greater extent, including glioblastomas (GBMs), breast carcinomas, epithelial ovarian carcinomas, and gastric carcinomas. The elucidation of the possible molecular mechanisms of AEP can contribute to the understanding of the diverse roles of AEP in the mentioned carcinomas and can facilitate the development of effective therapeutic strategies for breaking the therapeutic dilemma.

## 2. Glioblastoma

### 2.1. AEP Promotes Glioblastoma Progression by Blocking the Tumor-Suppressive Function of P53 Protein

Glioblastoma (GBM) is the most common and aggressive primary brain tumor in adults. Due to its different molecular features, GBM is divided in the WHO’s 2016 CNS into glioblastoma IDH-wildtype, glioblastoma IDH-mutant, and glioblastoma NOS. GBM IDH-wildtype corresponds most frequently with the clinically defined primary glioblastoma among patients over 55 years of age [44]. Despite the considerable progress achieved in neurosurgery, radiotherapy, and chemotherapy, the mean survival time of GBM patients is merely 12–15 months [45].

The observation that over half of human cancers have mutations in the P53 tumor-suppressive gene indicates the necessity of intact P53 activity for suppressing tumor development [46]. A majority of tumor types have high rates of missense mutations of the P53 gene; however, in most of GBMs, mutations in the P53 gene rarely occur, especially in IDH-wildtype GBMs [44,47,48,49,50,51].

**Figure 2:**
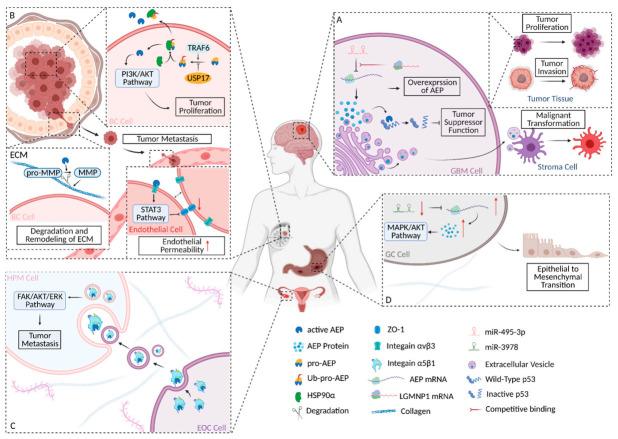
Schematic diagram of AEP’s roles in several carcinomas. (**A**) The biochemical and regulation mechanism of AEP in GBM. (**B**) The biochemical and regulation mechanism of AEP in BC. (**C**) Schematic diagram of the biochemical mechanism and involved signaling pathway of AEP in EOC. (**D**) Schematic diagram of the involved signaling pathway and regulation mechanism of AEP in GC. The figure was created using BioRender.com.

In 2020, Lin Y et al. reported a totally new way of regulating the activity of the P53 protein. Activated AEP cleaves P53 protein at N^311^, generating P53 fragments that have lost the function of transcriptional suppression of oncogenes, thus playing an indirect role in promoting the tumorigenesis, proliferation, and anti-apoptotic activity of GBM cells. Interestingly, the role of AEP in GBM is not limited to tumor cells. GBM cells secreted activated AEP into the tumor microenvironment (TME) in the form of extracellular vehicles (EVs). EVs are membranous nanoparticles that can be released by all cells to constitute an intercellular communication network. In the TME of GBM [52], where astrocytes are the predominant stroma cells, EVs containing large amounts of AEP stimulate the malignant transformation of normal astrocytes into reactive astrocytes (Figure 2A).

Another study on intercellular communication and GBM invasiveness could indirectly demonstrate the malignant transformation of astrocytes by AEP [53]. This study demonstrates that normal astrocytes exposed to GBM-EVs acquire a malignant phenotype through inhibition of the P53 pathway and activation of the MYC pathway. Although this study did not explore the main components of EVs, it is highly possible that AEP involves in the inhibition of P53 pathway and the activation of MYC pathway in normal astrocytes, thus presenting a reactive malignant phenotype that enhances the invasive ability of GBM cells. This hypothesis needs to be proven by further studies [53,54,55].

### 2.2. Modulation of AEP Expression in GBM

Like in other types of carcinomas, AEP is highly expressed in GBM. A report on AEP’s regulatory modality filled the gap in the upstream regulatory mechanism of AEP. A novel pseudogene, legumain pseudogene 1 (LGMNP1), acts indirectly to up-regulate AEP mRNA through competitive binding to miR-495-3p, leading to overexpression of AEP. Overexpression of AEP could enhance the invasive ability of GBM in vivo and in vitro [56,57] (Figure 2A).

### 2.3. Discussion about AEP’s Role in GBM

Preliminary validation of AEP as an effective target for tumor therapy was achieved with AEPI, which is the most promising chemotherapeutic agent for targeting AEP and AEP silencing by shRNA. Inhibition of AEP’s function can significantly suppress tumor progression and prolong the survival time of mice [53]. Due to its nature as a small molecule and its specificity, AEPI can effectively cross the blood–brain barrier (BBB) and has a promising future as a chemotherapeutic agent for the treatment of GBM.

Among the therapeutic strategies for targeting AEP, the exploitation of AEP as a prodrug activator has much had development for GBM. The principles involved in this strategy include the highly expressed AEP in tumor tissues and the tumor microenvironment, the strict restriction of AEP substrate, and the biocompatibility and designability of polymeric biomaterials [58].

Based on this principle, several amazing studies have been reported. Ruan S. et al. developed a nanoplatform, AuNPs-A&C, which allows passage through the BBB in GBM to increase nanoparticle tumor accumulation. By conjugating doxorubicin (DOX) and hydroxychloroquinea (HCQ), D&H-AuNPs-A&C became more effective in improving chemotherapeutic outcomes with the cotreatment of anti-PD-L1 antibody, which is a strategy for neutralizing immunosuppression in the TME of GBM [59,60,61].

In addition, Zhan J. et al. reported a nanomaterial named Comp.1 that enables the inhibition of GBM cell growth by disturbing lysosomal function with a similarly exploitation of the role of AEP in GBM [62].

A Kaplan–Meier analysis of AEP expression in 99 GBM cases showed that patients with low AEP expression had a median survival time of 26 months, while patients with high AEP expression had a median survival time of 8 months [53]. These results indicated that AEP is a potential biomarker that could be used to predict a poor prognosis. Although AEP mRNA levels were not prognostic in GBMs [53], the inclusion of AEP as a histochemical pathological index may guide clinical treatment.

## 3. Breast Carcinoma

Breast carcinoma (BC) is the leading cause of cancer-related deaths in women aged more than 45 years. The incidence and mortality rates of BC are expected to increase significantly in the next 5–10 years. The median survival in the metastatic field is dramatically low (~24 months) [63]. Several reports have shown that AEP is involved in tumor proliferation and metastasis, presenting diverse roles as a therapeutic target. Understanding the biochemical mechanisms of AEP in BC may enable the optimization of available targeted therapies and the development of combinations of treatments. Several reports have shown that AEP is involved in tumor progression and the TME of BC, presenting diverse roles as a therapeutic target.

### 3.1. Modulation of AEP in BC

The TME of BC involves kinds of stress stimulations, such as starvation and hypoxia, which enable the induction of expression of anti-stress proteins. As a reactive protein, tumor necrosis factor receptor-associated factor 6 (TRAF6) is a ubiquitination enzyme that mediates conjugation of Lysine-63(K63)-linked polyubiquitin chains to proteins. An early report showed that TRAF6 was involved in the regulation of pro-AEP in BC. Elucidating the regulation of pro-AEP can help in understanding the overexpression and cancer-promoting role of AEP in BC.

In BC cells, TRAF6 directly catalyzed K63-linked poly-ubiquitination of pro-AEP; this process could be reversed by USP17 [64]. Subsequently, heat shock protein 90-α (HSP90α), a stress-inducible molecular chaperone [65], was recruited by ubiquitinated pro-AEP to assemble complexes. The complexes enabled the maintenance of stabilization of pro-AEP and promoted the secretion of pro-AEP into the extracellular matrix under stress conditions [64]. On the one hand, stabilized pro-AEP can maintain a stable concentration of AEP in BC cells, thus playing a role in BC proliferation; on the other hand, in the TME, the secreted pro-AEP was activated to form AEP after a multi-step activation, thus playing its role in extracellular matrix remodeling and the mediation of tumor metastasis (Figure 2B).

### 3.2. AEP Promotes BC Progression Via the PI3K/AKT Pathway

A model of spontaneously developed mammary tumors in transgenic polyomavirus middle T antigen (PyVmT) mice was the perfect material for studies of the biochemical mechanism of AEP. In PyVmT mice, AEP was overexpressed in both primary tumor tissue and in lung metastasis tissue; injection of purified AEP significantly enlarged the tumor volume and increased lung metastases. Correspondingly, AEP inhibitor (AEPI) could effectively impede tumor volume increase and prolong the survival time of mice, thus confirming the feasibility of AEP as a therapeutic target in BC. Moreover, this report provides indirect evidence that AEP may promote tumor progression by regulating the activation of the AKT/PI3K pathway (Figure 2B).

### 3.3. AEP-Mediated Degradation and Remodeling of the Extracellular Matrix (ECM)

The tumor microenvironment is a robust influencing factor for the occurrence of tumor metastases [66]. In BC, not only was AEP secreted into the TME by tumor cells, but it was also expressed in stromal cells and tumor-associated macrophages (TAMs) [67]. The origin of AEP in surrounding cells of the TME in BC may be consistent with the origin of AEP in astrocytes of the TME in GBM, but this hypothesis requires experiments in order to be proven.

As a family of zinc-dependent endopeptidases, the central role of matrix metalloproteinases (MMPs) in the TME is the degradation and remodeling of the extracellular matrix (ECM). Of the MMP family, MMP-2 and MMP-9 acquire enzymatic activity through the cleavage of AEP. Activated AEP specifically cleaves the asparaginyl bond at the N-terminal of pro-gelatinases, producing a mature form of MMP-2 and MMP-9. Both of them are involved in the degradation of the ECM, which promotes the escape of BC cells in situ and the formation of a metastasis niche [68,69] (Figure 2B).

### 3.4. AEP-Mediated Tumor Metastasis through Increasing Endothelial Permeability

Tumor metastasis is a multifactorial event in BC. Kang L. et al. reported an intermediate role of AEP in increasing the permeability of endothelial barriers [70]. The endothelial tight junction is a blood–tumor barrier that suppresses tumor metastasis. As one of the tight junction proteins, zonula occludens 1 (ZO-1) induces the remodeling of the cytoskeleton to maintain the integrity of the tight junction under stress conditions. Due to the adaptive instincts of tumors, tumor cells secrete angiogenic factors to induce tumor angiogenesis [71,72]. Tumor-associated vessels could express a group of heterodimeric cell surface receptors named integrins, which are expressed less in the quiescent endothelium. Just like prisoners fabricate a key to escape from prison, endothelial cells induced by BC express integrin αvβ3 on their surface. Integrin αvβ3 could specifically recognize an Arg-Gly-Asp (RGD)-containing sequence [73]. The effect of integrin αvβ3 interaction with RGD is the closure of integrin αvβ3 [74]. Tumor-derived AEP harbors an RGD motif that can interact with endothelial integrin αvβ3. The closure of integrin αvβ3 indirectly down-regulates ZO-1 expression via the STAT3 signaling pathway, which finally increases the endothelial permeability in order to assist with the escape of the dissociated tumor (Figure 2B).

### 3.5. AEP Is an Effective Target of TME in BC

The highly expressed AEP in TAMs gives the TME an additional effective target. The therapeutic strategy of doxorubicin-based prodrug (leg-3), which is specifically activated by AEP, is notable. Leg-3 selectively “ablated” TAMs and resulted in a significant outcome in a mouse model. Treatment with the prodrug led to reduction of angiogenic factors and related tumor vessel growth, the suppression of circulating tumor cells and myeloid immune suppressor Gr-1+⁄CD11b+ cells, and the inhibition of tumor growth, exerting a combined antiangiogenic and antitumor effect [75].

Before understanding AEP’s role in TAM, we need to know that two major TAM polarization states exist in the TME: pro-inflammatory “M1” and pro-tumor “M2”. A study of the role of AEP in TAMs showed that the involvement of AEP in the TME polarization—performing as a depletion of AEP in TAMs—leads to the sustained activation of the JAK1/STAT1 signaling pathway, which was proposed to control the M1 phenotype. Subsequently, M1 elicits a tumor-inhibitory effect via induction of cellular senescence. The biochemical mechanism of the inhibitory effect is through the activation of the STAT1/iNOS/ROS axis; M1 secretes IL-1β, which mediates the senescence of BC [76].

Then, conversely, it can be inferred that in wild-type TAMs, AEP may mediate the M2 polarization of the TAMs and inhibit the M1 polarization of the TAMs by binding to integrin α5β1.

Overall, AEP in the TME can be taken into account in therapeutic strategies for targeting the TME, which can achieve suppression of immune evasion of tumors by the TME.

### 3.6. Discussion of AEP’s Role in BC

Overexpression of AEP is also a high-risk factor for the development of breast ductal carcinoma in situ (DCIS), but the underlying mechanisms promoting the transition from DCIS to invasive disease remain mysterious. The proteolytic properties of AEP and stromal degradation may be the answer to the riddle of DCIS. More evidence is needed to prove this hypothesis [77].

Similar with GBM, the level of serum AEP in breast cancer patients was proved to be positively correlated with poor prognosis, suggesting that it could also serve as a prognostic marker.

As a therapeutic target in BC, therapeutic strategies based on AEP mainly focus on (a) targeting AEP directly with a DNA vaccine or AEPI or (b) developing a chemotherapy-drug-based prodrug that is activated by AEP. Mai C. et al. reported a detailed review summarizing all therapeutic strategies for targeting AEP [43]. AEPI had good efficacy in limiting tumor growth and prolonging the survival time in mice.

Combined with the biochemical mechanisms of AEP described in our review, the following therapeutic strategies are proposed for consideration: (i) a combination of medications consisting of signaling pathway inhibitors and AEPI, which may improve the efficiency of chemotherapy; (ii) a multi-peptide DNA vaccine including AEP, TRAF6, and other potential target proteins; (iii) the development of prodrugs together with two chemotherapeutic agents.

## 4. Epithelial Ovarian Carcinoma

Epithelial ovarian carcinoma (EOC) is one of the most malignant and fatal carcinomas in the reproductive system. Unfortunately, because of the lack of comprehensive laboratory tests and specific symptoms in the early stages, more than half of EOC patients are diagnosed at advanced stages with widespread metastases. The peritoneal membrane is a key site for EOC progression and is involved in peritoneal dissemination, multiple organ metastases, refractory ascites, and retroperitoneal lymph node involvement [78]. In addition, due to the high recurrence rates, the overall five-year survival with EOC is still low [79]. Studies on the mechanism of peritoneal pre-metastases can help to diagnosis EOC at an early stage, thus facilitating the development of chemotherapeutic strategies for patients with peritoneal metastases.

### 4.1. AEP May Mediate Peritoneal Metastasis in Epithelial Ovarian Carcinoma

Similar to other carcinomas, AEP functionally enhances EOC progression in vitro and in vivo [80]. However, differently from other carcinoma types, AEP is highly expressed in both EOC and human peritoneal mesothelial cells (HPMCs). Integrin α5β1, which is specifically expressed in the EOC, is also highly expressed in both EOC and HPMCs; at the same time, integrin α5β1 and AEP are co-localized in both EOC and HPMCs.

Integrin belongs to a family of transmembrane cellular receptors that play a crucial role in the cellular communication of EOC cells and HPMCs through exosomes (which are also known as extracellular vesicles). Functionally, integrin α5β1 enables the recognition of the RGD sequence on fibronectin, one of the most abundant proteins in the extracellular matrix of the EOC peritoneum and omentum. It also enables binding with AEP in the same way [81,82]. According to the “seed-and-soil” hypothesis, integrin α5β1 assembles with AEP, making a complex in EOC cells. The integrin α5β1/AEP complex is secreted by EOC cells into ascites and serum in the form of exosomes. With the involvement of integrin α5β1, the exosomes derived from EOC cells are easily taken in by HPMCs; therefore, the cancer-promoting effect of AEP is brought into HPMCs (Figure 2C). This hypothesis has been tentatively confirmed in experiments.

The exosomal integrin α5β1/AEP complex may promote HPMC proliferation and migration via the FAK/AKT/ERK signaling pathway, and it is indirectly involved in the epithelial-to-mesenchymal transition (EMT) of peritoneal mesothelial cells [83].

### 4.2. Discussion of AEP’s Role in EOC

There is great potential for AEP to serve as an effective target in EOC due to its significant roles. The effect of AEPI’s targeting of AEP has been demonstrated in mice. Li X. et al. reported that AEP inhibitor can largely decrease the number of metastases in EOC models, which indicates that there is a great scope for developing therapeutic strategies.

Considering this review of the mechanisms in EOC, some ideas for the development of strategies for EOC patients with peritoneal mesothelial metastasis are provided: (i) a multi-peptide DNA vaccine that includes AEP and integrin α5β1; (ii) FAK/AKT/ERK pathway inhibitors combined with AEPI.

It was found in an analysis of clinical samples of the integrin α5β1/AEP complex that the level of the integrin α5β1/AEP complex was elevated in both the serum and ascites of patients, and it was positively correlated with poor prognosis. Likewise, this finding makes the integrin α5β1/AEP complex a prognostic predictor for EOC. Further exploration of the detected integrin α5β1/AEP complex in the early stages of EOC is needed in order to evaluate whether it could serve as a biomarker to help in the clinical screening of EOC patients.

## 5. Gastric Carcinoma

With over one million new cases in 2018, gastric carcinoma (GC) has ranked fifth in incidence, and thus has an enormous influence on human health. Tumor progression and metastasis are the primary causes of death in GC patients due to its advanced stage at the earliest diagnosis and poor prognosis. Especially in the treatment of GC patients with peritoneal metastasis, the lack of efficient molecular markers leads to the lack of early diagnoses and a decrease in the efficacy of radical resection, a widely used treatment method [84]. Although GC has not been studied in the field of AEP in as much detail and depth as BC, here, because of some similarities between the mechanisms identified in GC and those of BC, our review aims to provide researchers with ideas for the exploration of the role and biochemical mechanisms of AEP in GC.

### 5.1. Modulation of AEP in GC 

Zhang Y. et al. reported that GC patients with peritoneum metastasis have a low level of microRNA-3978 (miR-3978), which is significantly associated with highly expressed AEP protein. MiRNA is a class of short-stranded RNA that regulates post-transcriptional translation. Evidence shows that miR-3978 suppresses the expression of AEP by blocking the translation of AEP mRNA, rather than degrading AEP mRNA [85,86]. Meanwhile, in vitro and in vivo experiments demonstrated that dysregulation of AEP and miR-3978 is closely related to GC progression in situ and to distant metastasis. This report proposed the hypothesis that not can only AEP serve as a therapeutic target and biomarker, but miR-3978 may also enable prediction of peritoneum metastasis as a biomarker in GC (Figure 2D).

### 5.2. AEP May Promote GC Progression through Diverse Pathways

Previous reports showed that with highly expressed AEP, GC presents a feature of greater invasion and metastasis [87,88]. Cui Y. et al. reported a possible mechanism of AEP’s promotion of GC progression by modulating epithelial-to-mesenchymal transition (EMT). This conclusion was drawn by detecting changes in downstream signaling molecules after knocking down the AEP gene in GC cells. If AEP was knocked down, the expression level of the twist decreased significantly; the epithelial markers’ expression of the EMT and E-cadherin increased, but that of the mesenchymal markers, N-cadherin, β-catenin, and Vimentin decreased. In addition, if AEP was knocked down, the phosphorylation levels of the AKT and MAPK signaling pathways were significantly decreased, which indicated that the AKT and MAPK signaling pathways may be involved in the modulation. More in-depth and detailed studies are needed in order to explain the functional role of AEP in GC (Figure 2D).

Although coordinated expression of legumain and MMP-2 was observed in gastric cancer tissues, there is no direct evidence yet that AEP mediates the activation of MMP-2 in GC, as it does in BC [89]. In-depth investigation of this hypothesis could help reveal the commonality of AEP in these carcinomas.

### 5.3. AEP May Play a Crucial Role in Tumor-Associated Macrophages of GC

L.E. Edgington used an AEP-activity-based probe to prove that high levels of AEP expression were positively correlated with TAM activation [90]. Wang H. et al. reported that overexpression of AEP in TAMs may regulate GC cell proliferation and invasion in vivo and in vitro [91].

In the TME of GC, tumor-associated macrophages (TAMs) are vital factors that are presented at different stages of GC progression. TAMs may enhance vascular endothelial growth factor (VEGF) expression to promote angiogenesis and lymph-angiogenesis in GC [92]. In other aspects, TAMs confer cisplatin resistance to GC cells by activating the PI3K/AKT signaling pathway and down-regulating PTEN expression [93]. In tumor immunity, TAMs expressed CXCL8 to induce expression of PD-L1, which inhibited CD8^+^ T cell functions and eventually promoted GC progression [94]. Considering the significant effect of AEP on GC, it is worth exploring whether AEP is involved in the identified functional role of TAMs in GC.

### 5.4. Discussion of AEP’s Role in GC

As a potential target molecule for GC, treatment with AEPI could limit tumor growth and metastasis in mice models. In addition, other therapeutic strategies targeting AEP have rarely been reported. Referring to the strategy developed for BC, the following therapeutic strategies based on the role of AEP in GC can be considered: (i) a combination of medications consisting of signaling pathway inhibitors and AEPI is feasible; (ii) a prodrug activated by AEP can especially target tumors and the surrounding TME; (iii) immunotherapy plus AEPI is also feasible.

As a potential biomarker, AEP in GC is not limited in its use for predicting prognosis, but it enables the prediction of tumor metastasis at an early stage. This will greatly improve the efficacy of surgical treatment by intervening in tumor metastasis early, thus eventually improving the post-operative survival of clinical GC patients.

## 6. Conclusions

**Figure 3:**
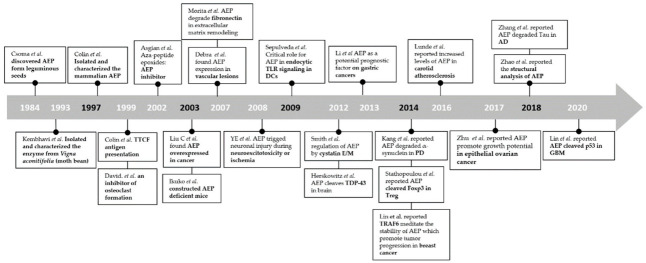
Timeline of basic research in the field of AEP.

In this review, particular emphasis was placed on highlighting the functions, mechanisms of action, and regulation of AEP, which will be beneficial to readers who are interested in protease in tumors, as well as scientists who are not currently working in this area. The feasibility of AEPI in the treatment of various types of carcinomas is also discussed. Meanwhile, as shown in Figure 3, we have also organized the major discoveries in the field of AEP according to the time of publication, so that readers can quickly know about the important research in the field of AEP.

To provide readers with a more visual understanding of the mechanism of AEP in carcinomas, four kinds of carcinomas that have been more intensively studied or are promising are summarized in Figure 2. The substrates and regulators of AEP in carcinomas are also summarized in Table 1.

## 7. Discussion

Discussions of targeting strategies and the effects of AEPI in four tumor types with respect to AEP were described in each section. Here, we provide a discussion on the unresolved issues concerning AEP.

The potential of AEP to serve as a target or prognostic factor has not only been reported in these four carcinoma types. Other carcinomas, including esophagus carcinoma [95], cervical carcinoma [96], uveal melanoma [97], colorectal carcinoma [98,99,100], prostate carcinoma [101,102], and urothelial carcinoma of the bladder [103], have been reported to involve AEP.

It is remarkable that in prostate carcinomas, AEP may also be involved in the activation of the PI3K/AKT signaling pathway, which is similar the role of AEP in BC [101]. It might be a common role of AEP in multiple carcinomas. Considering the promising role and specific enzymatic activity of AEP, the further studies of the functions and mechanisms of AEP in these carcinomas will be significant. A pending question in the field of AEP is that of what triggers the up-regulation of AEP, as well as how stress conditions of starvation or hypoxia induce up-regulation of AEP. The concrete signaling pathway regulation of the AEP protein and the activation of AEP in vivo are worth exploring. Figuring out the upstream mechanism of AEP in tumors will be helpful for blocking the carcinoma-promoting effect of AEP, and even predicting the early occurrence of tumors with “upstream” markers. Moreover, additional AEP substrates need to be identified in order to avoid adverse clinical events resulting from therapeutic strategies.

## Figures and Tables

**Table 1:** Substrates or regulators of AEP in the carcinomas.

Tumor Category	Substrate or Regulator	Effect	Reference
Glioblastoma	P53	Inhibition	[56]
	miR-495-3p	Inhibition	[64]
	LGMNP1	Expression Induction	[64]
Breast Carcinoma	Pro-MMP-2, -9	Activation	[69,70,71]
	integrin αvβ3	Inhibition	[43]
Epithelial Ovarian Carcinoma	integrin α5β1	Coactivation	[84]
Gastric Carcinoma	miR-3978	Inhibition	[88,89,90,91,92]
